# Supplementary material for: WordCommentsAnalyzer: A windows software tool for qualitative research
Source: F1000Res. 2018 Sep 4;7:536. Originally published 2018 May 3. [Version 2] doi: 10.12688/f1000research.14819.2 (PMC6137411; doi:10.12688/f1000research.14819.2)
Supplement: Supplementary file 3 [file f1000research-7-17614-s0002.tgz › b2a79496-4ada-47c4-9682-a99ce867836d.zip!/Eight Iranian Health Journals/Journal of Health System Research _33.docx]

© Journal of Health System Research . This work is licensed under CC BY-NC 4.0 (https://creativecommons.org/licenses/by-nc/4.0/).

Volume & Issue: دوره 8، شماره 6؛ 1391

Title: رویکردی نوین بر ارتباط الگوهای سالم غذایی در بیماری دیابت ملیتوس بر مبنای شواهد

http://hsr.mui.ac.ir/index.php/jhsr/article/view/330

مقدمه: رشد چشمگیر آمار مبتلایان به دیابت ملیتوس و خطر عوارض آن، لزوم تمرکز بر راه‌های کنترل دیابت از طریق اصلاح شیوه زندگی، الگوی غذایی و فعالیت بدنی را مطرح می‌نماید. این مطالعه مروری سیستماتیک به ارتباط الگوهای غذایی سالم بر کنترل هیپرگلایسمی (افزایش قند خون)، مقاومت به انسولین و دیابت ملیتوس نوع 2 می‌پردازد. روش‌ها: در این جستجو لغات کلیدی مورد بررسی مشتمل بر: الگوی غذایی Healthy، Prudent، Mediterranean و DASH (Dietary approach to stop hypertension)، Diabetes mellitus، Insulin resistance و Metabolic syndrome بود. جستجو در موتورهای جستجوی PubMed، ISI، Cochrane Central Register of Controlled Trials Databases، Iran Medex و Magiran اجرا شد. مجلات، کتاب‌های الکترونیکی، سمینارها، سمپوزیوم‌ها و مقالات (در قالب کوهورت آینده‌نگر، کارآزمایی بالینی تصادفی و مقطعی) منتشر شده بین سال‌های 1992 تا 2011 مورد بررسی قرار گرفتند. منابع بر اساس عنوان، خلاصه و متن کامل انتخاب شد. یافته‌ها: جمع‌بندی منابع مرتبط نشان داد که الگوی غذایی غنی از غلات کامل، آجیل، دانه‌ها، سبزیجات، میوه‌ها و فیبر و گوشت سفید بر خلاف الگوی خوراک سرشار از گوشت قرمز و فرآوری شده و غلات تصفیه شده دارای اثر حمایتی در کنترل هیپرگلایسمی است. الگوهای غذایی «سالم»، «مدیترانه‌ای»، «Prudent» و «DASH» با خطر کمتر دیابت ملیتوس نوع 2 همراه است. نتیجه‌گیری: تبعیت از الگوی غذایی سالم سرشار از غذاهای پرفیبر و فرآوری نشده، احتمال مقاومت به انسولین، هیپرگلایسمی و خطر دیابت نوع 2 را کاهش می‌دهد.

Title: سوء رفتار علمی: سرقت علمی از خود

http://hsr.mui.ac.ir/index.php/jhsr/article/view/690

سرقت علمی، یکی از مباحث جدی جامعه علمی محسوب می‌شود و در سال‌های اخیر، در کانون توجه و علاقه جامعه دانشگاهی و علمی قرار گرفته است. سرقت علمی از نظر محققین امری نادرست و غیر اخلاقی تلقی می‌شود؛ چرا که به منزله سرقت از کاری فکری است. اقتباس از آثار ادبی خود (Self plagiarism)، نوعی از سرقت علمی در نظر گرفته می‌شود و به عنوان ارایه نامناسب از اطلاعات منتشر شده خود به عنوان ایده‌ای جدید، اصلی و یا استفاده از قسمتی از مقاله منتشر شده قبلی خود تعریف شده است. در تعریف خود سرقت علمی به عنوان سرقت، استفاده نادرست یا تقلب و این که آیا شامل مجازات‌های سرقت علمی باشد یا خیر، هنوز تردیدهایی وجود دارد و در مطالعات متعدد به عنوان سرقت علمی معرفی شده است، اما در برخی از موارد مطالعاتی در رد این موضوع دیده می‌شود. همچنین از خود سرقت علمی تعاریف متعدد و گاه متفاوتی ارایه شده است. در مواردی به عنوان چاپ مجدد مقاله در مجله دیگر و یا چاپ با تغییر در عنوان و تغییرات جزیی به عنوان مقاله‌ای جدید مطرح شده است و گاهی به عنوان استفاده از عبارات، جملات و قسمتی از مقاله منتشر شده قبلی فرد بدون رفرنس‌دهی معرفی شده است. در مواردی نیز، به عنوان استفاده از ایده‌های قبلی و نه تنها استفاده از کلمات مطرح شده است. در هر حال، آن چه که مشخص است، خود سرقت علمی از سوی جامعه علمی مورد قبول نیست و نویسندگان در این مقاله بر این باورند که استفاده از آثار قبلی خویش، بدون رعایت ضوابط لازم و ارجاع درست، از مصادیق سرقت علمی می‌باشد و از این رو لازم است که محققین گرامی این مفهوم را تنها در استفاده نادرست از کلمات و جملات خلاصه نکنند و علاوه بر رفرنس‌دهی مناسب در استفاده از آثار خود، از ارایه ایده‌های قبلی در مقالات جدید و تقسیم داده‌ها برای انتشار در چند مقاله خودداری نمایند. این مطالعه با هدف ارایه و جمع‌بندی دیدگاه‌های مقالات منتشر شده از سوی نویسندگان و سردبیران مجلات داخلی و خارجی برای آشنایی محققین کشور به ویژه دانشجویان و محققین جوان انجام شد.

Title: خودکارآمدی در انجام فعالیت فیزیکی بیماران نارسایی قلبی: مقاله مروری

http://hsr.mui.ac.ir/index.php/jhsr/article/view/742

علایم فیزیکی بیماران نارسایی قلبی اغلب با کیفیت پایین زندگی این بیماران مرتبط است. تصور بر این است که بدتر شدن تدریجی در توانایی انجام فعالیت‌های روزانه و کاهش تحمل فعالیت فیزیکی منجر به افزایش نشانه‌ها در این بیماران می‌شود. فعالیت فیزیکی و ورزش می‌تواند ظرفیت عملکردی این بیماران را بهبود بخشد و نشانه‌های مربوط به بیماری را مرتفع نماید. به همین علت، ورزش به عنوان مکمل درمان‌های دارویی برای این بیماران توصیه می‌شود. در مطالعات گذشته، مداخلات مبتنی بر بهبود کارآمدی فرد مورد بحث بوده است. هدف از مطالعه مروری حاضر، این بود که به نقش خودکارآمدی به عنوان مهم‌ترین سازه تئوری شناختی اجتماعی و عوامل مؤثر بر آن در فعالیت فیزیکی بیماران نارسایی قلبی بپردازد.در ابتدا 5 پایگاه اطلاعاتی الکترونیکی برای شناسایی و جستجوی واژه‌های خودکارآمدی، نارسایی قلبی مزمن، نارسایی قلبی، و نارسایی احتقانی قلب و بازتوانی، فعالیت فیزیکی و ورزش در محدوده ماه اگوست 1992 تا 2012 استفاده شد. از نوشته‌های خاکستری و مروری نیز به منظور وارد نمودن تمام مطالعات مرتبط استفاده شد. بر اساس استراتژی جستجو و معیارهای در نظر گرفته شده، 12 مقاله در این مطالعه مورد بررسی قرار گرفت. مهم‌ترین عامل مؤثر برای انجام ورزش و فعالیت فیزیکی منظم بیماران نارسایی قلبی خودکارآمدی آنان است. علاوه بر اثر پیش‌گویی خودکارآمدی، این سازه در تبعیت بیماران از فعالیت فیزیکی نیز نقش مهمی را ایفا می‌کند. یافته‌های این مطالعه استراتژی‌های مهم برای افزایش خودکارآمدی بیماران و در نتیجه تبیعیت بیشتر آنان را انجام درست رفتار، تجربه جانشینی، ترغیب کلامی و برانگیختگی احساسات شناسایی کرد. تعدادی از مطالعات، ارتباط قوی خودکارآمدی با فعالیت فیزیکی انجام شده در بیماران نشان را دادند. شواهدی از چند مطالعه کارآزمایی نشان می‌د‌هد که ترکیب تئوری خودکارآمدی با طراحی مداخلات فعالیت فیزیکی در این بیماران سودمند است. علاوه براین، مداخلات فعالیت فیزیکی که مبتنی بر درک خود و خودکارآمدی ورزشی است، می‌تواند اثر بسزایی در اطمینان فرد از شروع آن و درنتیجه، بهبود علایم بیماری داشته باشد. در نهایت، چنین مداخلات خودکارآمدی باید به شکل گسترده‌تری به ویژه در تغییر رفتار بیماران مورد آزمایش قرار گیرد.

Title: بررسي تأثير آموزش خلاقيت مبتني بر روش Meeker بر ميزان توليد واگراي دانش‌آموزان استعدادهاي درخشان و عادي

http://hsr.mui.ac.ir/index.php/jhsr/article/view/359

مقدمه: تردیدی نیست که مهم‌ترین نقش آموزش و پرورش، رشد و پرورش توانایی خلاقیت در دانش‌آموزان است. Guilford خلاقیت را معادل با تولید واگرا می‌داند. وی نخستین کسی بود که مفهوم تولید واگرا را در روانشناسی خلاقیت مطرح کرد. او تولید واگرا را ویژگی اصلی خلاقیت می‌داند. تولید واگرا یکی از مفاهیم اساسی مربوط به عملیات در الگوی ساختار توانایی‌های شناختی Guilford است که بعدها توسط Meeker در زمینه ارزیابی آموزش مورد استفاده قرار گرفت. Meeker جهت سنجش توانایی‌های مربوط به تولید واگرا، خرده آزمون‌هایی ساخت. او همچنین برای تقویت و بهبود این توانایی‌ها، بسته‌های آموزشی را طراحی نمود که امروزه در مؤسسه ساختار توانایی‌های شناختی Meeker در لس‌آنجلس مورد استفاده قرار می‌گیرد. هدف این پژوهش، بررسی تأثیر آموزش خلاقیت مبتنی بر روش Meeker بر میزان تولید واگرای دانش‌آموزان مقطع راهنمایی شهر ایلام بود. روش‌ها: جامعه آماری، دانش‌آموزان دختر و پسر پایه‌های اول و دوم راهنمایی شهر ایلام در سال تحصیلی 89-1388 بودند. نمونه آماری، دانش‌آموزان پایه‌های اول و دوم راهنمایی دو مدرسه استعدادهای درخشان شهر ایلام و دو مدرسه راهنمایی عادی به تعداد 402 نفر بودند. تحقیق به روش نیمه آزمایشی و پیش آزمون- پس آزمون با گروه کنترل اجرا شد. از هر مدرسه 4 کلاس به صورت تصادفی به دو گروه آزمایشی و کنترل گمارده شدند. گروه آزمایشی به مدت 36 ساعت در معرض روش‌های آموزشی Meeker قرار گرفت و سپس پس آزمون برای هر دو گروه اجرا شد و تفاوت به دست آمده مشخص گردید. ابزار گردآوری داده‌ها، خرده آزمون‌های تولید واگرای Meeker بود. از آزمون تحلیل کواریانس استفاده شد. یافته‌ها: نتایج نشان داد که بین میزان خلاقیت دانش‌آموزان گروه‌های آزمایشی و کنترل در خرده آزمون‌های تولید واگرای تصویری، واحدهای معنایی و روابط نمادی تفاوت معنی‌داری وجود دارد. نتیجه دیگر تحقیق نشان داد که بین دانش‌آموزان استعدادهای درخشان و عادی از نظر میزان خلاقیت تفاوتی وجود ندارد. نتيجه‌گيري: نتايج اين تحقيق، لزوم توجه به موضوع خلاقيت و افزايش آن در ميان دانش‌آموزان را مطابق با بسته‌هاي آموزشي ميكر مورد تأیيد قرار مي‌دهد.

Title: بررسی رابطه هدف در زندگی، سبك‌هاي مقابله‌اي با بهداشت روانی در میان دانشجویان دانشگاه ایلام

http://hsr.mui.ac.ir/index.php/jhsr/article/view/450

مقدمه: همه ما انسان‌ها در زندگي به دنبال راهكاري جهت افزايش سلامت رواني هستيم، پس داشتن سلامت روان لازمه زندگي سالم است. بعضي معتقدند كه سلامت روان به معناي عدم اختلال رواني است. اما در روانشناسي مثبت‌نگر، به جاي تأكيد بر جنبه‌هاي آسيب شناختي، بر مسایلي چون اميد، خردمندي، خلاقيت، معنويت و تفكر درباره آينده و هدف در زندگي تأكيد مي‌شود. هدف این پژوهش تعیین ارتباط هدف در زندگی، سبک‌های مقابله‌ای با بهداشت روانی در میان دانشجویان دانشگاه ایلام بود. روش‌ها: 520 دانشجوی دختر و پسر به روش نمونه‌گیری طبقه‌ای متناسب انتخاب شدند. نوع مطالعه توصيفي- همبستگي بود. جمعيت هدف، دانشجويان دانشگاه ايلام در سال 88-1387 بودند. ابزار مورد استفاده در این پژوهش پرسش‌نامه راهبردهای مقابله‌ای Billings و Moos، پرسش‌نامه هدف در زندگی Majolica و Crum bough و پرسش‌نامه سلامت عمومی Goldberg بود. پایایی این ابزارها در مطالعات مختلف قابل قبول گزارش شده است. از آزمون‌هاي آماري ضريب همبستگي Pearson، تحليل واريانس يك‌طرفه، Turkey's test و آزمون t جهت تحليل داده‌ها استفاده شد. یافته‌ها: رابطه منفی معنی‌دار بین سبک مقابله‌ای هیجان‌مدار و سلامت روان مشاهده شد. همچنین بین متغیرهای سبک‌های مقابله‌ای، مسأله‌مدار و سلامت روان، هدف در زندگی و سلامت روان رابطه مثبت و معنی‌داری به دست آمد. نتایج تحلیل واریانس نشان داد که بین داشتن هدف در زندگی در رشته‌های مختلف، سلامت روان در رشته‌های مختلف، سبک‌های مقابله‌ای هیجان‌مدار در رشته‌های مختلف و سبک‌های مقابله‌ای مسأله‌مدار در رشته‌های مختلف، تفاوت معنی‌دار وجود دارد. نتایج حاکی از این بود که تفاوت بین دختران و پسران از نظر داشتن هدف در زندگی و سلامت روانی معنی‌دار نیست. اما تفاوت دختران و پسران در سبک‌های مقابله‌ای مسأله‌مدار معنی‌دار است. یعنی پسران از سبک مقابله‌ای مسأله‌مدار استفاده می‌کنند. نتیجه‌گیری: داشتن هدف و معنا در زندگی، نقش مهمی در پیشگیری از اختلالات روانی دارد. با عنایت به این که راهبردهای رویارویی مسأله‌مدار، نقش مهمی در سلامت روان دارند و باعث رضایت از زندگی می‌شوند، می‌توان با آموزش این راهبردها، به ایجاد یک زندگی سالم و رضایت‌بخش در افراد کمک نمود.

Title: ارتباط میان میزان مصرف روغن‌های گیاهی با سطوح آپولیپوپروتئین‌ها و چربی‌های سرم در ایرانیان

http://hsr.mui.ac.ir/index.php/jhsr/article/view/453

مقدمه: اثرات مضر روغن‌های گیاهی نیمه هیدروژنه بر روی آپولیپوپروتئین‌ها از چندین قسمت دنیا گزارش شده است، ولی اطلاعات اندکی از خاورمیانه در دسترس می‌باشد. این مطالعه با هدف، ارزیابی ارتباط میان نوع روغن‌های گیاهی، چربی‌ها و سطوح آپولیپوپروتئین‌های سرم در ایرانیان صورت گرفت. روش‌ها: در مطالعه مقطعی حاضر، اطلاعات 1772 فرد (795 مرد و 977 زن) 19 تا 81 ساله که به روش نمونه‌گیری تصادفی خوشه‌ای چند مرحله‌ای از سه شهرستان اصفهان، نجف آباد و اراک در «برنامه قلب سالم اصفهان» ( Isfahan healthy heart program یا IHHP) انتخاب شدند، استفاده شد. برای ارزیابی دریافت‌های غذایی معمول شرکت کنندگان، از پرسش‌نامه بسامد خوراک معتبر استفاده شد. مصرف روغن‌های گیاهی هیدروژنه (که برای پخت و پز در ایران استفاده می‌شوند) و مارگارین به عنوان گروه روغن‌های گیاهی نیمه هیدروژنه و مصرف روغن‌های سویا، آفتابگردان، ذرت، زیتون و کانولا به عنوان گروه روغن‌های گیاهی غیر هیدروژنه در نظر گرفته شدند. کلسترول (کل، LDL، HDL) سرم، تری‌گلیسرید و آپولیپوپروتئین‌های A و B به صورت ناشتا با روش‌های استاندارد اندازه‌گیری شدند. یافته‌ها: شرکت کنندگان با بالاترین دریافت روغن‌های گیاهی نیمه هیدروژنه و غیر هیدروژنه نسبت به کسانی که دریافت پایین‌تری داشتند، جوان‌تر بوده و وزن کمتری داشتند. مصرف بالای روغن‌های گیاهی نیمه هیدروژنه و غیر هیدروژنه با دریافت پایین‌تر انرژی، کربوهیدرات، فیبر غذایی و دریافت بالای میوه‌ها، سبزیجات، گوشت، شیر و غلات همراه بود. هیچ تفاوت معنی‌داری در سطوح آپولیپوپروتئین و چربی‌های سرم بین چارک‌های روغن‌های گیاهی غیر هیدروژنه و نیمه هیدروژنه پس از تعدیل مخدوشگرهای بالقوه دیده نشد. نتیجه‌گیری: هیچ ارتباط معنی‌داری بین دریافت روغن‌های گیاهی هیدروژنه و غیر هیدروژنه و سطوح آپولیپوپروتئین و چربی‌های سرم یافت نشد. مطالعات بیشتری برای بررسی این ارتباط نیاز می‌باشد.;

Title: شناسایی خطر با یک ابزار خاص؛ ارزیابی ریسک و مدیریت حرکات تکراری

http://hsr.mui.ac.ir/index.php/jhsr/article/view/567

مقدمه: تا به امروز، کامل‌ترین روش ارزیابی ریسک حرکات تکراری در اندام‌های فوقانی، روش OCRA (Occupational repetitive actions) می‌باشد. این شاخص علاوه بر آنالیز به نسبت دقیق عوامل خطر مختلف نظیر تکرار حرکات، نیرو، وضعیت بدنی، عوامل اضافی و تأثیرگذار و دوره‌های بازگشت، می‌تواند جهت اقدامات پیشگیرانه بعدی مفید باشد. مطالعه حاضر با هدف شناسایی خطر ناشی از ریسک فاکتورهای حرکات تکراری در اندام‌های فوقانی با استفاده از شاخص OCRA در مشاغل صنایع دستی استان اصفهان انجام گرفت. روش‌ها: در این مطالعه مقطعی، تلفیقی از چهار روش جمع‌آوری اطلاعات شامل روش مشاهده‌ای، مصاحبه، پرسش‌نامه Nordic و شاخص فعالیت‌‌های تکراری شغلی (OCRA) استفاده گردید. قلم‌زنی برجسته، قلم‌زنی مشبک، قلم‌زنی ساده، قلم‌کاری، میناکاری، کاشی‌کاری، تذهیب، خاتم‌کاری، مسگری و مینیاتور، مشاغل صنایع دستی موجود در شهر اصفهان را تشکیل می‌دهند. جامعه هدف شامل 94 نفر مرد بودند که به صورت تصادفی انتخاب گردیدند. جهت شناسایی خطر از پرسش‌نامه Nordic و جهت ارزیابی ریسک و مدیریت حرکات تکراری از شاخص OCRA استفاده گردید که در مجموع برای دست راست 94 وظیفه و برای دست چپ نیز 94 وظیفه مورد بررسی قرار گرفت. برای تحلیل داده‌ها از آزمون‌های آماری Chi-square، Independent-t، Kruskal–Wallis و آنالیز واریانس یک‌طرفه استفاده شد. یافته‌ها: اختلاف معنی‌داری بین میانگین شاخص OCRA در 10 شغل پیش‌گفت (001/0 > P) مشاهده گردید که این بیانگر پذیرش وجود ارتباط معنی‌دار بین سطح شاخص OCRA و نوع شغل (001/0 > P) می‌باشد. در این مشاغل سطح ریسک در دست راست به طور معنی‌داری بیشتر از دست چپ بود (001/0 > P)، که نشان دهنده عدم برابری شاخص OCRA در دست چپ و راست است. نتیجه‌گیری: میزان شیوع علایم اختلالات اسکلتی- عضلانی در کارگران مشاغل صنایع دستی مورد مطالعه بالا است. همچنین، سطح اقدام اصلاحی که بر اساس روش OCRA به دست آمد، گویای آسیب‌زا بودن شرایط و محیط کار در این حرفه است. بالاترین نمرات شاخص OCRA مربوط به وظیفه شغلی قلم‌زنی ساده ارزیابی شد و مشاغل خاتم‌کاری، قلم‌زنی برجسته، مسگری، قلم‌زنی مشبک، میناکاری، کاشی‌کاری، مینیاتور، تذهیب و قلم‌کاری به ترتیب در رتبه‌های ریسک پایین‌تری قرار گرفتند (001/0 > P).

Title: بررسی رفتار مصرف غذاهای سریع و عوامل مرتبط با آن در دانش‌آموزان مدارس راهنمایی شهر تهران در سال 1390: یک مطالعه مقطعی

http://hsr.mui.ac.ir/index.php/jhsr/article/view/572

مقدمه: امروزه تغییرات وسیع در شیوه زندگی سبب شده است که میزان استفاده از غذاهای سریع افزایش یابد. بنابراین با توجه به اهمیت تغذیه در سنین نوجوانی، در این مطالعه رفتار مصرف غذاهای سریع و عوامل مرتبط با آن در دانش‌آموزان مدارس راهنمایی شهر تهران مورد بررسی قرار گرفت. روش‌ها: مطالعه مقطعی (Cross-sectional) از نوع توصیفی- تحلیلی (Descriptive-analytic) حاضر با استفاده از پرسش‌نامه خودایفا بین 768 نفر (384 نفر پسر و 384 نفر دختر) از دانش‌آموزان پایه‌های اول، دوم و سوم راهنمایی در مناطق 2، 9 و 18 آموزش و پرورش تهران که با نمونه‌گیری تصادفی چند مرحله‌ای انتخاب شدند، انجام گردید؛ به طوری که در بین مناطق سه‌گانه منتخب، در هر یک از مناطق 2 و 9 دو مدرسه دولتی و دو مدرسه غیر انتفاعی راهنمایی پسرانه و دخترانه به طور تصادفی انتخاب شد و در منطقه 18 با توجه به تعداد کم مدارس غیر انتفاعی و کمیت دانش‌آموزان در این مدارس نمونه‌های مورد نظر در این منطقه از دو مدرسه دولتی انتخاب شدند. در مجموع نمونه‌های مورد مطالعه از ميان 10 مدرسه برگزیده شدند. داده‌های جمع‌آوری شده با استفاده از نرم‌افزار SPSS نسخه 18 مورد تجزيه و تحليل قرار گرفت. مشارکت افراد در این تحقیق داوطلبانه و با رضایت آگاهانه بود. یافته‌ها: به طور کلی 7/98 درصد دانش‌آموزان از غذاهای سریع استفاده می‌کردند که از میان آن‌ها 8/36 درصد 3-2 بار در هفته و 7/14 درصد هر روز غذای سریع مصرف می‌کردند. طبق یافته‌های این پژوهش، میزان مصرف غذاهای سریع در دانش‌آموزان پسر و دختر تفاوت معنی‌داری داشت (01/0 > P). نتایج حاصل از آزمون رگرسیون حاکی از این بود که جنسیت و منطقه سکونت دانش‌آموزان از عوامل تأثیرگذار در رفتار مصرف غذاهای سریع هستند. نتیجه‌گیری: نتایج حاکی از بالا بودن مصرف غذاهای سریع در دانش‌آموزان مدارس راهنمایی تهران بود. بنابراین انجام مداخلات آموزشی به منظور کاهش مصرف غذاهای سریع در جمعیت نوجوان کاملاً ضروری بوده و توصیه می‌شود.

Title: بررسی ارتباط بین تصور بدنی، خودکارآمدی و فعالیت فیزیکی در زنان شاغل در دانشگاه علوم پزشکی اصفهان و دانشگاه اصفهان

http://hsr.mui.ac.ir/index.php/jhsr/article/view/631

مقدمه:زندگی کم تحرک به عنوان مشکل جدی جامعه امروز ایران شناخته شده است. ارتقای سبک زندگی با افزایش میزان فعالیت فیزیکی در جهت سلامت زنان و پیشگیری از بیماری‌های قلبی- عروقی امری ضروری به شمار می‌رود. هدف از این مطالعه، بررسی ارتباط تصور بدنی، خودکارآمدی و فعالیت فیزیکی در زنان شاغل بود.   روش‌ها: مطالعه حاضر، مطالعه‌ای مقطعی از نوع توصیفی- تحلیلی بود که روی 408 نفر از کارمندان زن دانشگاه‌های علوم پزشکی اصفهان و دانشگاه اصفهان انجام گرفت. روش نمونه‌گيري به صورت سهمی متناسب با جمعيت بود. ابزار جمع‌آوری اطلاعات، پرسش‌نامه‌ای طراحی شده بر اساس پرسش‌نامه‌های استاندارد و خودساخته و شامل 4 بخش بود که متغیر‌های زمینه‌ای، رضایت بدنی، خودکارآمدی و فعالیت فیزیکی را در بر می‌گرفت. پس از جمع‌آوری اطلاعات، داده‌ها با استفاده از نرم‌افزار SPSS نسخه 18  به کمک آزمون‌های توصیفی و استنباطی مورد تجزیه و تحلیل قرار گرفت. سطح معنی‌داری 05/0 در نظر گرفته شد. یافته‌ها: به طور میانگین 6 /71 درصد زنان شرکت کننده در این پژوهش زندگی کم تحرک داشتند و فقط 4/28 درصد، سبک زندگی فعال داشتند. میانگین نمره رضایت بدنی در زنان مورد مطالعه 27/16 ± 26/63 (از 100) بود. ارتباط معنی‌داری بین نمره فعالیت فیزیکی زنان و BMI (Body mass index) دیده نشد؛ در حالی که ارتباط معنی‌داری بین فعالیت فیزیکی با رضایت بدنی و خودکارآمدی (01/0 > P) وجود داشت. بیشترین ارتباط بین رضایت بدنی با شرکت در کلاس‌های ایروبیک (001/0 > P) مشاهده شد. نتیجه‌گیری: به نظر می‌رسد زنان شاغل تحصیل کرده جامعه با وجود آگاهی از فواید فعالیت فیزیکی، سبک زندگی نامناسب دارند. شاید بتوان با مطالعات بیشتر در خصوص علل این مسأله و رفع موانع فعالیت فیزیکی، مثل ایجاد شرایط و تسهیلات بیشتر برای انجام فعالیت فیزیکی و بهبود فاکتورهای روانشناختی در محیط کار، به بهبود انجام فعالیت فیزیکی و ارتقای سلامت زنان که نقش به سزایی در سلامت خانواده و جامعه دارند، اهتمام ورزید.

Title: اثر مصرف چاي سبز بر درصد چربی بدن، شاخص‌های تن‌سنجی و فشار خون در زنان مبتلا به اضافه وزن و چاقي

http://hsr.mui.ac.ir/index.php/jhsr/article/view/647

مقدمه: طی دهه اخیر خواص ضد چاقی، کاهش خطر حمله قلبی و کاهش استرس اکسیداتیو چای سبز به طور وسیعی در مطالعات نشان داده شده است. هدف از این مطالعه، بررسی اثر مصرف چاي سبز بر درصد چربی بدن، شاخص‌های تن‌سنجی و فشار خون در زنان مبتلا به اضافه وزن و چاقي بود. روش‌ها: مداخله بالینی به روش Before-After در 41 زن مبتلا به اضافه وزن و چاقی به مدت 45 روز صورت گرفت. افراد در طول مطالعه روزانه 3 تا 5 فنجان (125 سی‌سی) چای سبز مصرف کردند. اطلاعات مربوط به ویژگی‌های دموگرافیک، شاخص‌های تن‌سنجی شامل قد، وزن، شاخص توده بدنی (Body mass index یا BMI)، درصد چربی بدن و همچنین فشار خون سیستولی و دیاستولی (در ابتدا و انتهای مطالعه) ثبت شد. یافته‌ها: میانگین سن، BMI و درصد چربی بدن به ترتیب 86/11 ± 18/38، 54/3 ± 86/28 و 51/3 ± 8/35 بود. میانگین فشار خون سیستول و دیاستول افراد شرکت کننده به ترتیب 20 ± 14/130 و 9/12 ± 8/84 میلی‌متر جیوه بود. در پایان مطالعه در میانگین شاخص‌های تن‌سنجی از جمله وزن، BMI و درصد چربی بدن کاهش معنی‌داری مشاهده نشد، اما فشار خون سیستولیک به طور معنی‌داری کاهش یافت (009/0 = P)، اگر چه فشار دیاستولیک تغییر معنی‌داری نشان نداد. نتیجه‌گیری: مصرف روزانه چای سبز، فشارخون سیستولیک را در زنان مبتلا به اضافه وزن و چاقی کاهش داد، اگر چه تغییر معنی‌داری در شاخص‌های تن‌سنجی حاصل نشد.

Title: بررسی مقایسه‌ای افسردگی، اضطراب و استرس در جوانان 18 تا 29 سال با رفتار و عدم رفتار سوء مصرف مواد

http://hsr.mui.ac.ir/index.php/jhsr/article/view/673

مقدمه: سوء مصرف مواد به عنوان یک رفتار مشکل مرتبط با سلامت مطرح است که کم و بیش تمامی کشورها با آن درگیرند. این رفتار دارای عوارض گسترده‌ای در سطح فردی و اجتماعی است که شناسایی عوامل مرتبط با گرایش به این رفتار را ضروری می‌نماید. مطالعه حاضر به مقایسه میزان افسردگی، اضطراب و استرس بین سوء مصرف کنندگان مواد و گروه مقایسه پرداخته است. روش‌ها: نوع مطالعه توصیفی- تحلیلی و از نوع مقایسه‌ای بود. جامعه مورد بررسی، جوانان با رفتار سوء مصرف مواد و عدم رفتار سوء مصرف مواد بودند. نمونه‌گیری به صورت تصادفی ساده و حجم نمونه در گروه سوء مصرف کنندگان مواد برابر با 183 و در گروه مقایسه برابر با 207 نفر بود. ابزار جمع‌آوری اطلاعات، پرسش‌نامه مشخصات فردی شامل 6 سؤال و پرسش‌نامه استاندارد 21-DASS (Depression Anxiety Stress Scale-21) که افسردگی، اضطراب و استرس را بازه زمانی گذشته می‌سنجد، بود. تجزیه و تحلیل داده‌ها با استفاده از آزمون‌های آماری Independent-t، Chi-square، Mann-Whitney و تحلیل Regression logistic انجام شد. یافته‌ها: میانگین سنی سوء مصرف کنندگان مواد برابر با 96/2 ± 9/25 سال و گروه مقایسه برابر با 36/3 ± 2/24 سال بود. بیشترین ماده مصرفی شیشه با 9/75 درصد بود. بین سطح تحصیلات دو گروه تفاوت معنی‌داری دیده شد (001/0 > P). نمره افسردگی، اضطراب و استرس در سوء مصرف کنندگان مواد بیشتر از گروه مقایسه و این تفاوت، معنی‌دار بود (001/0 > P). بر اساس نتایج تحلیل Regression logistic، اضطراب بیشترین همبستگی را با رفتار سوء مصرف داشت. نتیجه‌گیری: با توجه به ارتباط بین عوامل روانی و رفتار سوء مصرف مواد، ارایه برنامه‌های اجتماعی پیشگیری و درمان اختلالات روانی ضروری به نظر می‌آید.

Title: بررسي مقايسه‌ای علل سوء مصرف مواد مخدر از ديدگاه معتادين و خانواده آن‌ها (همراه) در مراجعين به مراكز ترك اعتياد شهرستان خرم‌آباد

http://hsr.mui.ac.ir/index.php/jhsr/article/view/680

مقدمه: با توجه به شیوع بالاي اعتیاد در کشور و مشکلات بهداشتی- اجتماعی ناشی از آن، اعتیاد به عنوان یکی از چند اولویت بهداشتی کشور محسوب می‌شود. این مطالعه با هدف، تعيين و مقايسه علل سوء مصرف مواد مخدر از ديدگاه معتادين و خانواده آن‌ها (همراه) در مراجعين به مراكز ترك اعتياد شهرستان خرم آباد انجام شد. روش‌ها: مطالعه حاضر به صورت مقطعي و بر روي معتادين و همراهان مراجعه کننده به مراكز ترك اعتياد دولتي شهر خرم آباد در محدوده سني 65-15 سال با نمونه‌گيري در دسترس و آسان انجام گرفت. ابزار مطالعه پرسش‌نامه‌ای مشتمل بر دو بخش بود که بخش اول شامل مشخصات دموگرافيک و علل سوء مصرف مواد مخدر در پنج بعد فردی، خانوادگی، اجتماعی، اقتصادی و روانی بود. اطلاعات جمع‌آوري شده با استفاده از نرم‌افزار SPSS نسخه 16 و آزمون‌هاي 2c، Mann-Whithney و Kruskal-Wallis مورد تجزيه و تحليل قرار گرفت. یافته‌ها: 3/48 درصد معتادین و 3/63 درصد افراد همراه شرکت کننده در مطالعه نقش اعتماد به نفس پایین را در اعتیاد مهم می‌دانستند. نیمی از افراد هر دو گروه اعتقاد داشتند که شکست‌های پیاپی در زندگی و یادگیری مشاهده‌ای از مصرف اعضای خانواده نقش شدیدی در اعتیاد دارد. از دیدگاه افراد همراه، نقش دو عامل نابرابری‌های اقتصادی و بی‌کاری و کمبود فرصت شغلی به طور معنی‌داری بیشتر از دیدگاه معتادین اهمیت داشت و درصد بیشتری از آن‌ها نقش دو عامل فوق را در اعتیاد شدید می‌دانستند، اما درصد بیشتری از معتادین، عواملی مانند اصرار دوستان و همسالان، تسکین درد و بیماری جسمی، احساس لذت در مصرف و نگرش مثبت فرد به مواد مخدر را زیاد می‌دانستند و این اختلاف دیدگاه بین دو گروه معنی‌دار بود (05/0 > P). نتیجه‌گیری: برای پیشگیری از اعتیاد و کاهش گرایش به مصرف مواد مخدر، اقداماتی اساسی در كاهش نگراني‌ها و اضطراب‌های اجتماعی، امنيت و رفاه اجتماعي، فرهنگ‌سازی سلامت به خصوص در حیطه روان و بهبود فضای اشتغال و اقتصاد جامعه ضروری است.

Title: بررسی تأثیر پمفلت آموزشی بر آگاهی و نگرش دانشجویان رشته‌های غیر پزشکی دانشگاه زابل در مورد بیماری ایدز

http://hsr.mui.ac.ir/index.php/jhsr/article/view/683

مقدمه: با توجه به اهمیت جوانان در گسترش و خطر ابتلا به ایدز و از آن‌جا که تنها یکی از ابزارهای مهم کنترل بیماری آموزش می‌باشد، این مطالعه به منظور ارزیابی تأثیر پمفلت آموزشی بر آگاهی و نگرش دانشجویان رشته‌های غیر پزشکی دانشگاه زابل در مورد بیماری ایدز طراحی گردید. روش‌ها: این مطالعه نیمه تجربی از نوع قبل و بعد با روش نمونه‌گیری تصادفی ساده بر روی 80 نفر از دانشجویان غیر پزشکی دانشگاه زابل انجام شد. پرسش‌نامه خودساخته بر اساس اهداف تهیه و روایی محتوایی توسط تعدادی از اعضای هیأت علمی صاحب نظر تأیید گردید. پرسش‌نامه در دو مرحله قبل و 40 روز بعد از مداخله تکمیل شد. داده‌ها توسط نرم‌افزار SPSS نسخه 18 با استفاده از آزمون‌های آماری Independent t و Paired t و ANOVA تجزیه و تحلیل شدند. یافته‌ها: میانگین نمره آگاهی بعد از آموزش از 76/7 به 15 از 16 نمره و نمره نگرش از 45/6 به 06/10 از 11 نمره افزایش یافت. با استفاده از آزمون آماری Paired t، 005/0 > P می‌باشد. نتیجه‌گیری: از آن‌جایی که تأثیر پمفلت آموزشی بر ارتقای آگاهی و نگرش دانشجویان تأیید گردید، با توجه به پایین بودن نمرات آگاهی در دانشجویان غیر علوم پزشکی توصیه می‌شود، برنامه آموزشی مستمر در مورد راه‌های انتقال و پیشگیری از بیماری ایدز به کلیه دانشجویان در سایر دانشگاه‌های غیر علوم پزشکی ارایه گردد.

Title: تأثیر آموزش مهارت‌های زندگی بر عزت نفس و کاهش خشونت خانگی علیه زنان شهر گرگان

http://hsr.mui.ac.ir/index.php/jhsr/article/view/700

مقدمه: نیاز به عزت نفس یکی از نیازهای روانی و طبیعی انسان است. آموزش مهارت‌های زندگی به زنان کمک می‌کند تا خود را بهتر بشناسند و بتوانند با رفتارهای خشونت‌آمیز علیه خود به درستی کنار آیند. هدف پژوهش حاضر، بررسی تأثیر آموزش مهارت‌های زندگی بر عزت نفس زنان تحت پوشش کمیته امداد امام خمینی شهر گرگان در پیشگیری از خشونت خانگی در سال 1390 بود. روش‌ها: مطالعه حاضر یک تحقیق نیمه تجربی با طرح پیش‌آزمون و پس‌آزمون بود که با حضور 91 نفر از زنان تحت پوشش کمیته امداد امام خمینی شهر گرگان در سال 1390 انجام شد. نمونه‌های پژوهش به صورت نمونه‌گیری تصادفی آسان انتخاب شدند. ابزار جمع‌آوری اطلاعات، پرسش‌نامه استاندارد عزت نفس Rosenberg و پرسش‌نامه دموگرافیک بود. مداخله آموزشی در 3 جلسه 120 دقیقه‌ای با روش بحث گروهی و توزیع جزوه آموزشی برگزار شد. 45 روز بعد از مداخله آموزشی، پس‌آزمون برگزار گردید و داده‌ها با استفاده از نرم‌افزار کامپیوتری SPSS نسخه 16 و روش‌های آمار توصیفی و استنباطی مورد تجزیه و تحلیل قرار گرفت. یافته‌ها: میانگین نمره عزت نفس زنان قبل آموزش از 29/5 به 664/6 بعد از مداخله آموزشی تغییر نمود، بدین معنی که آموزش اثر معنی‌داری بر ارتقای عزت نفس واحدهای مورد پژوهش داشت. تحلیل نتایج با استفاده از آزمون Independent t روی تفاضل میانگین نمره‌های پیش‌آزمون و پس‌آزمون نشان داد که آموزش مهارت‌های زندگی تأثیر معنی‌داری بر افزایش نمره عزت نفس دارد (001/ > P). با کمک آزمون رگرسیون خطی (Linear regression) ارتباط بین آموزش مهارت‌های زندگی و عزت نفس پس از مداخله آموزشی معنی‌دار بود و رابطه معنی‌دار و مستقیمی بین آن‌ها دیده شد. ارتباط بین میزان خشونت نیز قبل از مداخله آموزشی نسبت به بعد از مداخله معنی‌دار بود. نتیجه‌گیری: با آموزش مهارت‌های زندگی می‌توان به ارتقای سطح سلامت روانی و افزایش عزت نفس در زنان رسید. آموزش مهارت‌های زندگی سبب کسب تجربه، حل مسأله و ارتباط مؤثر در زنان می‌گردد که مانع از بروز رفتارهای منفی از جانب زن در منزل می‌شود و این خود افزایش اعتماد به نفس و کاهش خشونت را به همراه دارد.

Title: اثر بخشی راهبردهای شناختي- رفتاري آموزش مدیریت استرس در زنان مبتلا به اختلال افسردگی

http://hsr.mui.ac.ir/index.php/jhsr/article/view/753

مقدمه: مطالعات مختلف نشان می‌دهد که با گسترش فشار روانی بر جامعه، تعداد مبتلایان به اختلالات روانی به ویژه افسردگی افزایش می‌یابد و این امر سبب می‌شود، اختلال افسردگی بخش چشمگیری از شکایات مددجویان را تشکیل دهد. مطالعه حاضر با هدف، بررسی اثربخشی آموزش مدیریت استرس بر اساس راهبردهای شناختی- رفتاری بر میزان استرس و افسردگی زنان مبتلا به اختلال افسردگی در شهر اصفهان انجام شد. روش‌ها: در مطالعه حاضر ۴۰ بیمار مبتلا به اختلال افسردگی از مراکز و کلینیک‌های مشاوره و روان‌پزشکی شهر اصفهان از طریق نمونه‌گیری چند مرحله‌ایی انتخاب و به صورت تصادفي در دو گروه مداخله و شاهد (20 نفر در هر گروه) تقسيم شدند. آموزش مدیریت استرس در گروه مداخله به مدت 8 جلسه 90 دقیقه‌ای به شيوه شناختي- رفتاري انجام پذیرفت. ابزار جمع‌آوری اطلاعات شامل پرسش‌نامه فشار روانی Cooper و پرسش‌نامه افسردگی Hamilton بود که قبل و یک ماه پس از مداخله در هر دو گروه تکميل شد. داده‌هاي حاصل با استفاده از آزمون ANCOVA تجزیه و تحلیل گردید و برای همه آزمون‌ها سطح معنی‌داری 05/0 در نظر گرفته شد. یافته‌ها: پس از مداخله آموزشی کاهش معنی‌داری در میانگین نمرات استرس و افسردگی افراد در گروه مداخله نشان داده شد (001/0 > P). تفاوت بين ميانگين‌هاي تعديل شده دو گروه در پس‌آزمون، در مورد فشار روانی (45/12 = F، 001/0 > P) و افسردگی (36/5 = F، 02/0 = P) نیز معنی‌دار بود. نتیجه‌گیری: آموزش مدیریت استرس بر اساس راهبردهای شناختی- رفتاری به بیماران مبتلا به اختلال افسردگی، با افزایش توانایی بیماران در برخورد با رویدادهای استرس‌زای زندگی ضمن کاهش فشار روانی سبب بهبود علایم افسردگی گردید.

Title: بررسی کارایی نانولوله‌های کربنی چند جداره اصلاح شده با هیپوکلریت کلسیم در حذف بنزن از محلول‌های آبی

http://hsr.mui.ac.ir/index.php/jhsr/article/view/762

مقدمه: بنزن يک ترکيب فرار و تک حلقه‌اي آروماتيک است و در محصولات نفتي وجود دارد که مهم‌ترین نگراني در مورد آن خاصیت سرطان‌زایی بنزن می‌باشد. هدف از این مطالعه، بررسی قابلیت حذف بنزن با استفاده از نانولوله‌های کربنی چند دیواره و نوع اصلاح شده آن با هیپوکلریت کلسیم و هیپوکلریت سدیم از محلول‌های آبی بود. روش‌ها: در مطالعه حاضر غلظت بنزن 100 میلی‌گرم بر لیتر، غلظت نانولوله‌هاي کربني 5/0 گرم بر لیتر و زمان تماس 1 ساعت و 2 ساعت و pH خنثي براي انجام آزمايش در نظر گرفته شد. نمونه‌هاي مورد نياز به صورت شبيه‌سازي شده در آزمايشگاه ساخته شد و توسط دستگاه گاز کروماتوگرافي جرمي آناليز گرديد. یافته‌ها: ظرفيت جذب (qe) و راندمان حذف (R) برای بنزن توسط نانولوله‌هاي کربني چند دیواره اصلاح شده با هیپوکلریت کلسیم (mg/g 6/188 = qe و 3/94 درصد = R) بيشتر از نانولوله‌هاي کربني چند ديواره اصلاح شده با هیپوکلریت سدیم (mg/g 7/187 = qe و 9/93 درصد = R) و نانولوله‌هاي کربني چند دیواره اصلاح نشده (mg/g 6/182 = qe و 3/91 درصد = R) مي‌باشد. نتیجه‌گیری: نانولوله‌های کربنی اصلاح شده با هیپوکلریت کلسیم می‌تواند به طور مؤثري بنزن را از آب جذب نمايد و داراي پتانسيل خوبي براي تصفيه آب و فاضلاب‌هاي آلوده به مواد نفتي است.

Title: سازگاری شناختی بیماران دیابتی نوع 2 مراجعه کننده به بیمارستان‌های تابعه دانشگاه علوم پزشکی تهران در تبعیت از درمان

http://hsr.mui.ac.ir/index.php/jhsr/article/view/792

مقدمه: دیابت نوع 2 به عنوان شایع‌ترین بیماری متابولیک با عوارض فراوان است و تبعیت از درمان در بهبود پیامدهای سلامتی این بیماران نقش به سزایی دارد. اين پژوهش، با هدف بررسی سازگاری شناختی در تبعیت از درمان در بیماران دیابتی نوع 2 و تعیین عوامل مؤثر بر این سازگاری طراحی و انجام شد. روش‌ها: اين پژوهش يك مطالعه توصیفی- تحلیلی بود كه در سال 1391 انجام شد. جامعه پژوهشی در این مطالعه، بیماران دیابتی نوع 2 مراجعه کننده به بیمارستان‌های تابعه دانشگاه علوم پزشکی تهران‌، حجم نمونه 480 نفر و نمونه‌گيري به روش تصادفی ساده با نمونه‌های در دسترس انجام شد. سازگاری شناختی در تبعیت از درمان بیماران دیابتی نوع 2 با استفاده از پرسش‌نامه دو قسمتی شامل اطلاعات دموگرافیک و مرتبط با بیماری و سازگاری شناختی بر اساس مقیاس پنج درجه‌ای لیکرت اندازه‌گیری شد. داده‌های جمع‌آوری شده با استفاده از نرم‌افزار SPSS نسخه 5/11 با استفاده از آزمون‌های 2χ و مدل رگرسیون لجستیک مورد تجزیه و تحلیل قرار گرفت. سطح معنی‌داری در این مطالعه 05/0 بود. یافته‌ها: جنسیت (001/0 = P)، وضعیت تأهل (001/0 > P)، سال‌های ابتلا به بیماری دیابت (001/0 > P)، وضعیت شغلی (001/0 > P)، سطح تحصیلات (001/0 > P)، سابقه خانوادگی مثبت دیابت (001/0 = P)، گروه‌های سنی (001/0 > P)، وضعیت درآمد (001/0 > P)، بیماری‌های زمینه‌ای همراه (02/0 = P) و HbA1C (001/0 > P) با سازگاری شناختی در تبعیت از درمان دیابت ارتباط آماری معنی‌داری داشتند. در مدل رگرسیون لجستیک هم عوامل سن، وضعیت تأهل، درآمد، سابقه ابتلا به دیابت، وجود بیماری‌های زمینه‌ای همراه و سال‌های سپری شده با دیابت و HbA1C شانس سازگاری بالا و معنی‌داری داشت. نتیجه‌گیری: با عنایت به تأثیر عوامل دموگرافیک و بالینی در میزان سازگاری شناختی در تبعیت از دیابت، ضرورت انجام مداخلات ارتقای سلامت مبتنی بر تئوری‌های سازگاری اجتناب‌ناپذیر بود.

Title: بررسی تأثیر اشعه UV-C بر حذف بنزن از جریان هوا برای مقادیر مختلف رطوبت در فرایند UV/O3

http://hsr.mui.ac.ir/index.php/jhsr/article/view/814

مقدمه: بنزن یکی از پرکاربردترین ترکیبات آلی فرار است که اثر مخربی بر سلامتی انسان دارد. حذف و یا کنترل بنزن از هوا، یکی از موضوعات مورد بحث در مهندسی محیط زیست است. هدف از این مطالعه، اثربخشی اشعه UV-C (Ultra violet-C) در هوای مرطوب بر چگونگی حذف بنزن از جریان هوا در فرایند uv/o3 بود.  روش‌ها: مطالعه حاضر از نوع تجربی و در مقیاس آزمایشگاهی انجام شده است. سیستم آزمایش شامل پمپ هوا، پمپ تزریق سرنگی، محفظه اختلاط، روتامتر، ژنراتور تولید ازن، هیتر، ایمپینجر و رآکتور استیل به طول cm 45 و حجم مفید 35/1 لیتر که در داخل آن یک لامپ 15 وات UV-C با طول موج 254 نانومتر قرار داده شده بود. غلظت‌های مشخص بنزن به صورت مداوم در زمان ماندهای متفاوت و رطوبت‌های مختلف در معرض فرایند uv/o3 قرار گرفت. میزان غلظت بنزن قبل و بعد از در معرض قرارگیری با متغیرهای ذکر شده سنجش گردید. داده‌ها با استفاده از آمار توصیفی و استنباطی و به کارگیری آزمون Three- way ANOVA (Three- way analysis of variance) مورد تجزیه و تحلیل قرار گرفت. یافته‌ها: نتایج نشان داد که با افزایش رطوبت نسبی تا 60 درصد اثربخشی UV-C (Ultra violet-C) در حذف بنزن سیر صعودی دارد و در رطوبت هوای بیش از 60 درصد، از تأثیر آن کاسته شده است. همچنین یافته‌ها حاکی از آن بود که با افزایش زمان واکنش در حضور اشعه UV-C و رطوبت بیشترین مقدار حذف بنزن (2/13 درصد) در جریان هوا اتفاق می‌افتد. نتیجه‌گیری: با توجه به ارتقای راندمان حذف بنزن در هنگام استفاده توأم از اشعه UV و رطوبت و نتایج آزمون Tukey در خصوص تأثیر افزایش زمان واکنش در موقع کاربرد همزمان اشعه UV و رطوبت نسبی می‌توان نتیجه‌گیری کرد که استفاده از اشعه UV-C در هوای حاوی رطوبت 60-50 درصد، اثر هم‌افزایی مثبت در حذف بنزن دارد (001/0 < P).

Title: بررسی موانع حمایتی و سازگاری زندگی با دیابت و عوامل مؤثر بر آن‌ها در بیماران دیابتی نوع 2 مراجعه کننده به بیمارستان‌های تابعه دانشگاه علوم پزشکی تهران

http://hsr.mui.ac.ir/index.php/jhsr/article/view/825

مقدمه: ابعاد روانی و رفتاری از موارد مهم زندگی با دیابت محسوب می‌شود. این مطالعه به منظور بررسی موانع حمایتی و سازگاری در زندگی با دیابت و عوامل مؤثر بر آن‌ها در بیماران دیابتی نوع 2 مراجعه کننده به بیمارستان‌های تابعه دانشگاه علوم پزشکی تهران طراحی و اجرا شد. روش‌ها: این مطالعه از نوع مقطعی (توصیفی- تحلیلی) بود که در سال 1391 بر روی 600 بیمار به طور تصادفی ساده انجام شد. ابزار مطالعه شامل دو بخش اطلاعات دموگرافیک و مرتبط با سلامت و بیماری و شاخص کنترل دیابت (HbA1C) با 10 سؤال و ابزار زندگی با دیابت در دو مانع حمایتی (8 سؤال) و سازگاری (8 سؤال) بود که توسط مقیاس از کاملا موافقم (5) تا کاملا مخالفم (1) اندازه‌گیری گردید. روایی و پایایی ابزار اندازه گرفته شد. روش جمع‌آوری اطلاعات، تکمیل پرسش‌نامه با استفاده از مصاحبه بود. برای تجزیه و تحلیل داده‌ها از نرم‌افزار آماری SPSS نسخه 5/11و آزمون‌های آماری توصیفی (میانگین و انحراف معیار) و استنباطی (2χ و مدل رگرسیون لجستیک) استفاده شد. سطح معنی‌داری در این مطالعه کمتر از 05/0 در نظر گرفته شد. یافته‌ها: میانگین و انحراف معیار موانع حمایتی مرتبط با دیابت و سازگاری به ترتیب 80/3 ± 71/22 و 68/5 ± 87/24 بود که متغیرهای جنس (01/0 = P)، سال‌های ابتلا به بیماری دیابت (001/0 > P)، سطح تحصیلات (001/0 > P)، نوع درمان (001/0 > P)، وضعیت شغلی (001/0 > P)، گروه‌های سنی (001/0 > P)، وضعیت درآمد (001/0 > P)، سابقه خانوادگی مثبت دیابت (001/0 > P) و HbA1C (001/0 > P) با موانع حمایتی مرتبط با دیابت ارتباط آماری معنی‌داری داشت. از طرف دیگر، نتایج نشان داد که متغیرهای سال‌های ابتلا به بیماری دیابت (001/0 > P)، سطح تحصیلات (001/0 > P)، نوع درمان (001/0 > P)، وضعیت شغلی (001/0 > P)، گروه‌های سنی (001/0 > P)، وضعیت درآمد (001/0 > P)، سابقه خانوادگی مثبت دیابت (001/0 > P) و HbA1C (001/0 > P) با موانع سازگاری با دیابت ارتباط آماری معنی‌داری داشت. نتیجه‌گیری: نتایج مطالعه حاکی از این است که عوامل متعددی در ابعاد سازگاری و حمایتی با بیماران ارتباط دارد، متغیرهای مرتبط با کلاس اجتماعی و سابقه ابتلا با شانس شدید بودن مانع حمایتی و سابقه ابتلا، درآمد و HbA1C سال‌های ابتلا به دیابت با شانس شدید بودن مانع سازگاری ارتباط دارد که این امر می‌تواند در طراحی و برنامه‌ریزی مداخلات ارتقای سلامت کمک کننده باشد.

Title: شناسایی گونه باکتری‌های تجزیه کننده هیدروکربن‌ها در خاک‌های آلوده به نفت خام اهواز

http://hsr.mui.ac.ir/index.php/jhsr/article/view/993

مقدمه: استخراج بلند مدت و تولید مشتقات نفتی متنوع باعث گسترش آلودگی در خاک‌های اطراف مکان‌های استخراج و پالایش نفت می‌شود. بزرگ‌ترین نگرانی در این مورد، خطرات زیست محیطی این آلاینده‌ها می‌باشد. هدف مطالعه حاضر، شناسایی و جداسازی باکتری‌های بومی تجزیه کننده آلاینده‌های هیدروکربنی نفت در خاک‌های آلوده منطقه اهواز بود. روش‌ها: بدین منظور از خاک‌های آلوده اطراف چاه‌های نفت مارون اهواز یک نمونه مرکب خاک تهیه شد و یک نمونه خاک غیر آلوده نیز از مناطق فوق تهیه و به شکل مصنوعی با نفت خام در سطح 5 درصد آلوده گردید. شمارش باکتری‌های تجزیه کننده نفت به روش بیش‌ترین تعداد احتمالی (Most probable number یا MPN) صورت گرفت و در مرحله بعد غنی‌سازی و خالص‌سازی باکتری‌های رشد کرده بر روی محیط آگار مغذی انجام شد. از تست‌های افتراقی و رنگ‌آمیزی گرم برای شناسایی خانواده و یا جنس گونه‌های جداسازی شده استفاده گردید. یافته‌ها: تعداد باکتری‌های تجزیه کننده در خاک آلوده طبیعی 105 × 8/6 و در خاک آلوده به روش مصنوعی برابر با 107 × 5/3 شمارش گردید. نتیجه‌گیری: نتایج جداسازی نشان داد که در خاک آلوده طبیعی باکتری‌های تجزیه‌گر شامل جنس‌های استافیلوکوکوس، اسینتوباکتر و فلاووباکتریوم هستند و در خاک با سطح آلودگی 5 درصد باکتری‌های تجزیه‌گر متعلق به جنس‌های اسینتوباکتر، سودوموناس و مورکسلا می‌باشند.
